# Cardiac Repair and Mesenchymal Stem Cells: Exploring New Frontiers in Regenerative Medicine

**DOI:** 10.2174/011573403X376065250728094646

**Published:** 2025-08-01

**Authors:** Sadia Nazir, Tahir Maqbool, Sumeyra Savas

**Affiliations:** 1 Institute of Molecular Biology and Biotechnology (IMBB), University of Lahore, Lahore, Pakistan;; 2 Department of Clinical Microbiology, Medical School, Bandırma Onyedi Eylül University, Balikesir, Bandırma, Turkey

**Keywords:** Stem cell, cardiac repair, regenerative medicine, mesenchymal stem cells, cardiovascular disease, repair mechanisms

## Abstract

Cardiovascular diseases, especially myocardial infarction, remain the prominent causes of death globally, necessitating the exploration of innovative therapeutic strategies. Medical and surgical available treatments mainly manage disease symptoms and prevent deterioration, but do not focus on the repair of lost cardiomyocytes. Mesenchymal stem cells (MSCs) have emerged as a promising tool for heart repair and regeneration after injury, as they possess unique properties, such as the potential for differentiation into cardiomyocytes and vascular endothelial cells, immunomodulation, the release of mediators, and paracrine effects. This review focuses on the latest understanding of MSC therapies for cardiac repair, specifically addressing their properties, mechanism of action, preclinical and clinical studies, problems and prospects, and future strategies. MSCs can be isolated from various tissues, including bone marrow and adipose tissue, each with its own advantages and disadvantages in cardiac repair. Many preclinical studies conducted concluded that MSCs could differentiate into cardiomyocytes. MSCs involve multiple factors that enhance angiogenesis, promote the survival of existing myocardium and cardiomyocytes, reduce fibrosis, modulate the immune response, activate existing cardiac stem cells, and facilitate tissue remodeling; all of these processes are crucial in myocardial repair after MI. Although preclinical studies have promising outcomes, the application of MSC therapy in clinical trials has faced many challenges. Clinical trials conducted so far have yielded variable outcomes, with some showing marked improvements and others producing no promising results, indicating less improvement in cardiac function and mortality. This variability may be due to multiple sources, including MSCs, delivery methods, culture conditions, the timing of administration after MI, and patient-dependent factors, such as disease severity, overall patient well-being, and other comorbid conditions. The review concluded that although MSCs have a significant role in cardiac repair, further research is essential for overcoming current challenges to unlocking the maximum regenerative potential of these cells.

## 1 INTRODUCTION

This review focuses on the latest understanding of MSC therapies for cardiac repair, specifically addressing their properties, mechanism of action, preclinical and clinical studies, problems and prospects, and future strategies. After a comprehensive literature search, relevant studies were identified by systematically searching the following databases using the terms MSCs, stem cell regeneration, stem cell therapy, and cardiovascular diseases: PubMed, Scopus, Web of Science, and Google Scholar. The search included peer-reviewed original research articles, systematic reviews, and meta-analyses. Editorials, conference abstracts, case reports, unpublished theses, and studies not related to the main objectives of the review were excluded from the search. Study titles and abstracts were manually screened, and studies were identified that were more relevant to the core topic of the review. Main data points, including study objectives, methodology, key findings, and conclusions, were retrieved and summarized. The data were thematically grouped to integrate existing knowledge and identify converging and conflicting perspectives. No formal meta-analysis was performed; however, a critical evaluation and interpretative summary were used to ensure a true reflection.

Cardiovascular diseases, specifically ischemic heart disease (IHD), are the primary causes of morbidity and mortality globally, with a significant burden on the health care system. Myocardial infarction (MI) is an irreversible myocardial injury due to the complete closure of one or more coronary arteries, leading to myocardial ischemia. After myocardial ischemia, a permanent injury is developed, having dead and dying cells, which is known as an infarct. Due to reduced blood flow in the infarcted area, this area becomes hypoxic, resulting in failure of mitochondrial function and a shift from aerobic to anaerobic energy generation. This shift leads to increased utilization of glucose and a higher production of lactic acid, which causes a drop in pH below 6.5. At this low pH, extensive hypoxia-induced death of cardiomyocytes occurs [1]. Monocytes, macrophages, and neutrophils migrate to the area of infarction, and an inflammatory response is produced. This initiates the signal transduction process for regulating cardiac repair, ultimately leading to the formation of permanent avascular fibrous scar tissue. These changes result in reduced heart function, which can lead to heart failure and death [2].

Early detection of the disease leads to a better prognosis. Despite the availability of common cardiac biomarkers, recent studies have focused on developing new diagnostic tools to improve the accuracy of disease diagnosis. A study showed that circulating miR-19a acts as a potential biomarker for early detection and prognosis of MI [3]. Another study demonstrated that microRNA-1 and microRNA-221-3p levels are upregulated in myocardial infarction and correlate with the levels of common cardiac markers, such as cardiac troponin I, creatinine, and creatine kinase-myocardial band, in patients with myocardial infarction [4].

Medical and surgical treatments of MI mainly focus on relieving symptoms, slowing the progression of the condition, and improving quality of life. However, they cannot repair or revert the changes due to MI. Only a heart transplant is a treatment option for an advanced disease with compromised cardiac function. However, due to the scarcity of donors and the expense of transplant operation, it is not considered the treatment of choice. Thus, with the emergence of regenerative medicine, the human heart has become a primary focus due to its limited capacity for self-regeneration and repair. Recent studies reveal that the human heart has the potential for repair [5-7]; however, this ability is significantly reduced after MI due to extensive loss of heart muscle. This loss can cause an overload on surviving cardiac muscles and potentiate the risk of heart failure [8].

## 2 MSC THERAPY AND MI

Therefore, novel and effective treatment strategies are needed to enhance cardiac repair, promote angiogenesis, reverse myocardial infarction (MI)-induced changes, and improve cardiac function. Stem cell therapy has emerged as an innovative and promising approach for the treatment of cardiac diseases, particularly in heart regeneration and the repair of damaged tissue following MI [9].

Mesenchymal stem cells (MSCs), a type of multipotent stem cell, have become one of the most attractive candidates for repairing injured myocardial tissue. MSCs can be isolated from various sources, with the most common being the umbilical cord, bone marrow, and adipose tissue [10]. They resemble fibroblasts morphologically and can be expanded in culture, which makes them suitable for use in both preclinical and clinical trials [11].

According to the International Society for Cellular Therapy (ISCT), MSCs possess the capacity for self-renewal and can differentiate into multiple cell types, including bone, cartilage, fat, and muscle, particularly cardiac muscle. They must adhere to plastic surfaces in culture and express specific positive surface markers such as CD29, CD44, CD73, CD90, CD106, and CD166, while lacking or minimally expressing negative markers like CD11b, CD14, CD19, CD3, CD45, and/or HLA-DR [12, 13].

MSCs, also known as medicinal signaling cells according to some authors, are recognized for their ability to secrete a wide range of soluble immunomodulatory and trophic factors. These factors act *via* autocrine, paracrine, and endocrine mechanisms on the damaged heart, enhancing its restorative capacity. MSCs promote angiogenesis, mobilization of resident stem cells, and cardiomyogenesis, thereby contributing to cardiac repair [14].

In addition, MSCs possess anti-fibrotic and anti-inflammatory properties. They lack markers for Major Histocompatibility Complex class II (MHC II), CD40, and CD80/86, which allows them to evade immune rejection [15]. MSCs can be derived from both allogeneic and autologous sources. Clinical trials have shown that MSCs from either source, when administered to patients with myocardial infarction, result in favorable ventricular remodeling, a beneficial immune response, and improved ejection fraction [16].

Several studies have described immunomodulation as a key mechanism by which MSCs prevent MI-induced adverse cardiac remodeling. This immunomodulatory activity is stimulated by specific inflammatory mediators and growth factors [17-20].

## 3 MSC-MEDIATED CARDIAC REPAIR

**Fig. (1):**
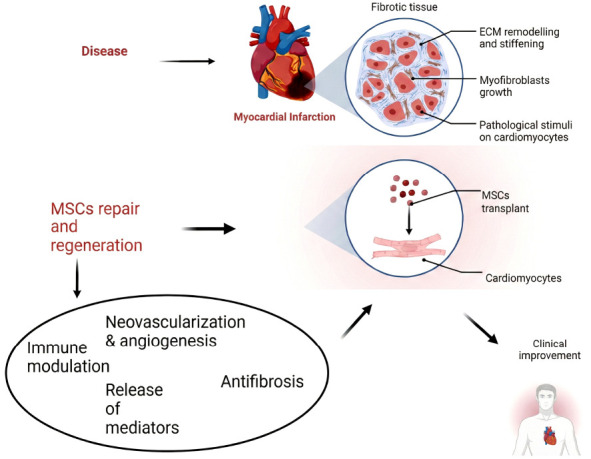
MSCs mediated cardiac repair *via* direct and indirect mechanisms.

The precise mechanism by which MSCs facilitate cardiac repair remains unclear. However, several mechanisms have been proposed, including implantation and differentiation into cardiomyocytes, the action of paracrine mediators released by MSCs, stimulation and proliferation of endogenous cardiac stem cells, neovascularization, and immunomodulation (Fig. **1**).

## 4 EFFECT OF CULTURE CONDITIONS ON MSC DIFFERENTIATION INTO CARDIOMYOCYTES

The criteria for MSC differentiation into cardiomyocytes include the expression of cardiac-specific markers, the presence of functional properties such as spontaneous beating, the formation of gap junctions, and the exclusion of cell fusion events [21]. The implantation and differentiation potential of MSCs are highly dependent on culture conditions. A variety of culture media, differing in animal serum concentrations and growth or differentiation factors, can influence outcomes.

Key culture conditions, such as oxygen concentration, cell density, genetic and epigenetic modifications, and passage number, significantly affect MSC preparation and differentiation potential [22]. Culturing MSCs under low-oxygen conditions activates the Akt signaling pathway, leading to the overexpression of vascular endothelial growth factor (VEGF) and ultimately enhancing cell viability, proliferation, and angiogenesis, thereby improving transplant outcomes [23].

Both *in vitro* and *in vivo* studies have demonstrated that MSCs can differentiate into cardiomyocytes when treated with agents, such as 5-azacytidine, as reported by Farag *et al*. [24]. Furthermore, when MSCs are exposed to a combination of bone morphogenetic protein-2 (BMP-2), fibroblast growth factor-4 (FGF-4), and hepatocyte growth factor (HGF), they can differentiate into cardiomyocyte-like cells and express cardiac-specific markers, such as troponin T (TnT), α-actinin, and transcription factors like GATA4 and NKx2.5 [25-27].

Studies have focused on enhancing the engraftment efficiency of MSCs following their infusion into the injured heart, with strategies, such as genetic engineering, aimed at improving the homing, survival, and function of these cells [28-30]. Research conducted in rat models has shown that genetic modification of MSCs to overexpress Akt (a kinase involved in cell survival) and Bcl-2 (an anti-apoptotic gene), while downregulating Bax (a pro-apoptotic gene), significantly enhances the survival of MSCs both *in vivo* and *in vitro*. This results in improved cardiac function and inhibition of adverse remodeling in myocardial infarction (MI) models [29, 30].

In addition, basic fibroblast growth factor (bFGF) has been reported to promote MSC differentiation into cardiomyocytes and to improve both homing and survival of MSCs in damaged cardiac tissue [31-34]. Furthermore, a study by Shen *et al*. demonstrated that the overexpression of miRNA-1-2 can induce MSC differentiation into cardiomyocytes by upregulating the expression of cardiac-specific markers, such as GATA4, NKx2.5, and cardiac troponin I (cTnI), *via* activation of the Wnt/β-catenin signaling pathway [35].

## 5 ROLE OF PARACRINE MEDIATORS RELEASED FROM MSCS

One of the major factors in repairing heart tissue is the effect of paracrine mediators released by MSCs. Sid-Otmane *et al*. (2020) initially described the paracrine effects of MSCs as primarily due to the secretion of numerous bioactive molecules collectively known as the secretome [32]. This secretome includes growth factors, cytokines, and extracellular vesicles (EVs), which transport proteins, lipids, and genetic material to recipient cells. Furthermore, the composition of secretomes can be modulated by preconditioning MSCs during cell culture.

Despite the relatively small number of implanted or retained MSCs, their paracrine signaling can facilitate reparative effects, including vasculogenesis, apoptosis prevention, and mobilization of resident cardiac stem cells. Key factors secreted by MSCs, including vascular endothelial growth factor (VEGF), transforming growth factor (TGF), and matrix metalloproteinases (MMPs), specifically MMP-2 and MMP-14, regulate angiogenesis and enhance endothelial cell survival [36-40].

Extracellular vesicle secretion represents one of the major paracrine mechanisms employed by MSCs. EVs are a heterogeneous group of cell-derived, membrane-bound vesicles that serve as vehicles for bidirectional cellular communication. Through their paracrine effects, MSCs promote angiogenesis *via* the synergistic action of various EVs carrying bioactive molecules such as microRNAs (miRNAs), transfer RNAs (tRNAs), growth factors, and proteins [40].

Similarly, EVs derived from BM-MSCs demonstrate enhanced proliferation and migration of fibroblasts, thus improving cardiac repair by promoting angiogenesis through endothelial cell tube formation. Angiogenesis is mediated through various pathways, such as the signal transducer and activator of transcription 3 (STAT3), which regulates VEGF expression [41]. Activation of the Akt/Nrf2 pathway is essential for enhancing the angiogenic role of VEGF secreted by MSCs. Additionally, overexpression of Akt maintains normal pH in the myocardium after MI, which is crucial for the survival of myocardial cells post-MI [42]. miR-19a derived from MSCs’ EVs causes overexpression of GATA4, thereby enhancing survival through its cardioprotective role and promoting angiogenesis. Meanwhile, miR-210 decreases the expression of Efna3, further promoting angiogenesis in MI models [43].

The antifibrotic activity of MSCs is also paracrine-mediated, as suggested by previous studies that show hepatocyte growth factor (HGF) released from MSCs prevents cardiac fibrosis after MI. Studies have shown that after the transplantation of MSCs into the damaged infarcted area, the released HGF acts *via* two mechanisms: one through direct cellular contact and the other by miR-155-mediated inhibition of signals promoting fibrosis, thereby enhancing cardiac remodeling and improving function in the MI model [44].

Due to the paracrine effects of MSCs, the growth and differentiation of cardiac stem cells (CSCs), which predominantly reside in specialized niches in the atria and apex of the heart, are stimulated. The stimulation and proliferation of endogenous CSCs by MSCs in cardiac regeneration have garnered considerable attention. One study reported that MSCs enhance the growth and differentiation potential of CSCs. The c-kit+ CSC clusters, originating from the heart itself, were detected in MSC-treated MI-damaged hearts, with more than 90% expressing the NKx2.5 cardiac differentiation marker [45, 46].

## 6 IMMUNOMODULATORY EFFECT OF MSCS

MSCs exhibit numerous biological characteristics, including broad differentiation potential, low immunogenicity, the ability to regulate immune responses, and the ability to release bioactive molecules that support the remodeling of damaged tissue. These qualities make them highly attractive candidates for cell-based therapy. Among these features, the immune-evasive capability of MSCs stands out as particularly important in cardiac immunobiology. This ability is largely due to their low expression of MHC class I and absence of MHC class II molecules.

To improve MI treatment outcomes, further research is needed to explore and enhance the immunomodulatory and immune-evasive properties of MSCs. The immunomodulatory functions of MSCs have been well documented in previous studies and are considered a key mechanism in mitigating adverse post-MI cardiac remodeling [47]. However, immunomodulation is not an intrinsic property of MSCs; rather, it requires activation by inflammatory mediators. Following MI, damage-associated molecular patterns (DAMPs) trigger the activation of tissue-resident macrophages, which subsequently release pro-inflammatory mediators [48].

These chemical mediators trigger immune responses, such as the respiratory burst in neutrophils, leading to their activation and degranulation, followed by the release of reactive oxygen species (ROS), proteases, and various other inflammatory mediators. This cascade initiates a strong immune response, contributing to vascular endothelial and myocardial damage.

Cardiac fibroblasts play a crucial role in producing and depositing collagen, which leads to the formation of permanent scars. Their activity is influenced by pro-inflammatory cytokines, such as TNF-α, IL-1, and IL-6, which are primarily secreted by M1 macrophages in the myocardium following myocardial infarction (MI). The healing process after MI requires a delicate balance between clearing necrotic debris and regulating scar tissue formation. Depletion of macrophages after MI disrupts this balance, impairing collagen deposition, hindering necrotic tissue clearance, limiting angiogenesis at the injury site, and increasing the risk of cardiac rupture.

**Fig. (2):**
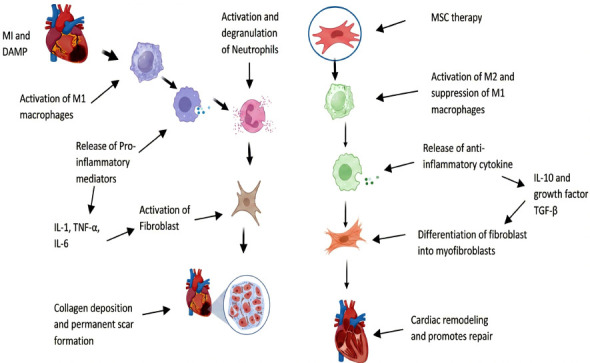
Immunomodulatory effect of mesenchymal stem cells.

In contrast, M2 macrophages secrete anti-inflammatory mediators, including IL-10 and TGF-β. These cytokines facilitate the differentiation of fibroblasts into myofibroblasts, ultimately supporting cardiac remodeling and repair. Investigating the molecular pathways that regulate the balance between the pro-inflammatory (M1) and reparative (M2) roles of macrophages may serve as a crucial strategy for MSC-mediated modulation of post-MI heart remodeling and may accelerate the cardiac repair process (Fig. **2**) [49].

Dendritic cells (DCs) are specialized immune cells that function as phagocytic antigen-presenting cells. They act as a crucial bridge between innate and adaptive immune responses. In many co-culture experiments, MSCs regulate and influence the functions, behavior, development, and maturation of DCs; their migration to the lymph node is also inhibited, resulting in a decreased *in vivo* allo-stimulatory capability of T cells. Furthermore, activated MSCs inhibit the proliferation of T cells by directly influencing the adaptive immune system, as noticed in a mixed lymphocyte reaction. This suppression is achieved through various soluble factors, including indolamine 2,3-dioxygenases, PGE2, nitric oxide (NO), TGF, and HGF [50]. MSCs not only suppress the proliferation of T cells but also influence their development and differentiation. As observed in an allograft rat model, the longevity of transplanted heart cells is enhanced when MSCs are injected simultaneously *via* both intrathymic and intravenous routes. This increased viability was linked to a change in the equilibrium of Th1/Th2, along with enhanced differentiation of CD4+, CD25+, and Foxp3+ regulatory T cells, and a decrease in IL-2 and interferon-gamma (IFN-γ) levels. A concordant increase in the levels of anti-inflammatory cytokines IL-4 and IL-10 was recorded [51]. MSCs inhibit T-cell activity and proliferation, consequently affecting B-cell modulation, which involves antibody-producing cells that highly depend on T cells. Studies showed that B-cell behavior is influenced by human MSCs, especially when MSCs are pre-treated with IFN-γ. The main effect is on direct inhibition of B-cell proliferation, differentiation, and chemotactic behavior. In an allogenic co-culture experimental study, B-cells were found to be halted in the G0/G1 phase of the cell cycle, with markedly reduced production of IgM, IgG, and IgA antibodies. There was a downregulation of CXCR4, 5, and 7 receptors. Based on this evidence, it is evident that MSCs have a significant and wide-ranging effect on the immune system, with the potential for immune modulation and homing to injured areas [51, 52]. This makes MSCs a carrier for generated soluble factors, which play a protective role during cardiac injury. Moreover, MSCs have the potential to produce anti-fibrotic and matrix metalloproteinases (MMPs), which cause modification in the extracellular matrix. This quality is crucial in treating chronic heart disease, especially scar remodeling, and thus leads to reperfusion and rigidity, enhancing cardiac regeneration.

## 7 PRECLINICAL STUDIES ON MSC THERAPY

MSCs have been utilized in several preclinical studies. Animal models have been commonly used in MSC transplantation studies on cardiac tissue to investigate the impact on cardiac function, repair mechanisms, and regenerative processes in conditions, such as myocardial infarction, acute myocarditis, and cardiomyopathies. The primary focus is to assess the fate of transplanted MSCs, their interaction with the surrounding tissue, their effect on the site of injury, and ultimately, the animal's response in terms of recovery and potential side effects. A few pre-clinical studies conducted on MSC transplantation to injured hearts are summarized in Table **1** [53-63].

These are a few exemplary studies that primarily focus on examining the efficacy of MSC transplantation and MSC-derived exosomes in promoting cardiac repair and improving cardiac function *via* various mechanisms, such as reducing inflammation, enhancing angiogenesis, improving tissue remodeling, and reducing fibrosis after injury. Due to the high prevalence rate of MI, animal models of various sizes have been utilized in studies to demonstrate the functional benefits and adverse effects [64].

## 8 CLINICAL TRIALS ON MSC THERAPY

Many clinical trials have been carried out on injured hearts to evaluate the effectiveness of MSC transplantation from both autologous and/ or allogenic sources in treating cardiac injuries. Some completed trials are mentioned in the (Table **2**) [65-68].

These studies yielded a wide range of outcomes, including improvements in heart function and reductions in scar tissue size, though some showed non-significant changes. Despite the promising results of MSC transplantation, including reduced scar size, enhanced local blood flow and contractility, promotion of angiogenesis, decreased fibrosis in injured tissue, and improved quality of life, several limitations remain in clinical trials. These include variability in cell dose, timing and mode of delivery, cell processing methods, and, most importantly, inadequate long-term follow-up.

## 9 STANDARDIZED PROTOCOLS FOR MSC THERAPY

For the effective utilization and production of MSCs in clinical practice for cardiovascular diseases, it is essential to establish standardized protocols and ensure compliance with Good Manufacturing Practices (GMP) [69]. Before clinical application, the safety, feasibility, and reproducibility of the techniques must be clearly demonstrated. The production of MSCs using well-defined protocols is of paramount importance, particularly when transitioning from preclinical studies to clinical settings. Therefore, there is a pressing need to develop standardized procedures for the isolation, expansion, and characterization of MSCs.

To meet GMP standards, cell culturing should be conducted in a tightly controlled environment that closely approximates a closed system [70]. Compliance with GMP criteria also requires that every step of the procedure is clearly defined and thoroughly documented. This includes details on the source tissue, methods of cell segregation and/or enrichment, cell seeding density, and the composition and conditions of the culture media, including the use of specific serums (*e.g.,* fetal calf or human), cytokines, and growth factors [71].

## 10 SAFETY, EFFECTIVENESS, AND FEASIBILITY OF MSC THERAPY

The efficacy, safety, and feasibility of MSC-based therapy for cardiovascular diseases (CVDs) have been demonstrated through numerous in-depth studies. MSCs show significant promise, particularly for myocardial infarction (MI) therapy. However, several challenges remain. The heterogeneity and complexity of MSC sources, along with the lack of standardized culture protocols, contribute to inconsistent therapeutic outcomes. MSCs derived from bone marrow, adipose tissue, and umbilical cord differ in their differentiation potential and immunoregulatory properties when applied in CVD contexts. Moreover, MSC characteristics, including viability and immunomodulatory capacity, are influenced by culture conditions such as oxygen levels, serum composition, and passage number [72-74].

Despite these differences, MSCs must meet key criteria, including the expression of specific cell surface markers and the ability to differentiate into adipogenic, osteogenic, and chondrogenic lineages. Their therapeutic efficacy largely depends on successful engraftment and survival at the transplantation site. However, the percentage of MSCs that survive post-transplantation is typically low. Factors such as the ischemic microenvironment can affect their immunogenicity and viability, often resulting in only modest improvements in cardiac function. Experimental studies indicate that a substantial number of MSCs are lost shortly after administration into the injured myocardium [75]. Therefore, further advancements are essential to improve MSC survival rates and fully optimize their therapeutic potential in cardiac repair.

Furthermore, MSCs' safety and effectiveness over time are still being explored, specifically focusing on the risk of arrhythmogenesis and tumorigenesis. According to a recent study, MSCs have a crucial protective role against the development of arrhythmias [76]. Another study conducted in 2023 showed a decrease in both simple and complex arrhythmias after MSC therapy [77]. With the recent developments in genetics and biomedical engineering, new opportunities for optimizing the therapeutic potential of MSCs have emerged. Overexpression of growth-promoting genes and downregulation of apoptotic genes enhance cardiomyocyte function and survival in a challenging post-MI environment. A recent study concluded that genetic manipulation with CXCR4 and Brachyury overexpression enhances MSC survival, engraftment, and reparative activity after myocardial infarction [78]. Moreover, tissue engineering techniques, like the utilization of hydrogels and scaffolds, create a conducive microenvironment that augments cell retention, engraftment, and survival [78-80]. Certain factors influence the outcomes of MSC therapy, such as age, comorbidities, and genetic predispositions. Age significantly impacts MSC function, with aged MSCs exhibiting reduced immunosuppressive potential and a shift toward a pro-inflammatory phenotype [81]. Other factors affecting MSC therapy outcomes include sex, biological source, and genetic predispositions [82].

## CONCLUSION

Mesenchymal stem cells (MSCs) offer a broad range of advantages, including their diverse sources, the relative simplicity of their isolation and expansion, and their low immunogenicity. Furthermore, when MSCs are implanted in affected regions, they possess the unique ability to migrate to areas of infarction. This enables them to exhibit immunomodulatory and anti-fibrotic responses. In addition, MSCs play a crucial role in promoting and facilitating angiogenesis, the formation of new blood vessels, and have the capacity to differentiate into cardiomyocytes. Collectively, these properties contribute to the repair of damaged myocardial tissue following a myocardial infarction.

Nevertheless, despite the wide range of advantages MSCs offer in treating cardiovascular diseases, several challenges remain. These include inefficient migration and homing to affected areas, as well as a very low survival rate under ischemic conditions, especially following myocardial infarction. The feasibility and safety of MSC therapy have been evaluated in numerous clinical trials; however, further extensive research is still needed. Future studies should focus on identifying optimal dosage levels, enhancing the anti-inflammatory properties of MSCs, exploring combinations with biomaterials, optimizing delivery routes, and developing standardized protocols for MSC differentiation into cardiomyocytes to improve treatment efficacy for myocardial infarction. Despite these ongoing challenges, the use of MSCs for cardiovascular diseases remains a promising and valuable approach within cell-based therapies.

## Figures and Tables

**Table 1:** Summary of preclinical studies on MSC transplantation in injured hearts.

**Study**	**Model**	**Conclusion**	**Authors**	**Publication Year**
Intravenous MSC-derived exosomes	Dilated cardiomyopathy (Rat)	Reduced myocardial inflammation	Sun *et al*.[53]	2018
Engineered exosomes	Myocardial infarction (Mouse)	Enhanced targeted therapy	Wang *et al*.[54]	2018
MSCs and exosomes	Cardiac repair (Mouse)	Shared mechanisms to enhance cardiac repair *via* miRNA	Shao *et al*. [55]	2017
MSC-derived exosomes	Ischemia-reperfusion injury (Mouse)	Decrease injury through miR-182-mediated polarization of macrophages	Zhao *et al*. [56]	2019
GATA-4-expressing MSCs	Myocardial infarction (Rat)	Improved cardiac function through the secretion of exosomes	He *et al*. [57]	2018
Akt-modified MSC-derived exosomes	Myocardial infarction (Mouse)	Improved cardiac regeneration and promoted angiogenesis	Ma *et al*. [58]	2017
MSCs overexpressing MIF	Myocardial repair (Rat)	Enhanced myocardial repair	Liu *et al*. [59]	2020
Ischemic preconditioning of MSCs	Myocardial infarction (Rat)	Potentiated protective effects through the release of exosomes targeting Mecp2 *via* miR-22	Feng *et al.* [60]	2014
Intramyocardial MSC injection	Myocardial infarction (Porcine)	Improvement of cardiac function and reduction in the size of scar tissue	Makkar *et al*. [61]	2005
Bone marrow-derived MSCs	Myocardial infarction (Rat)	Significant improvements in post-infarction heart function	Hare *et al*. [62]	2009
MSCs enhancing cardiac repair	Myocardial infarction (Mouse)	Promoted angiogenesis and reduced apoptosis	Hatzistergos *et al*. [63]	2010

**Table 2:** Summarization of clinical trials on MSC therapy in CVDs.

**Study**	**Authors**	**Publication Year**	**Study Outcome**
POSEIDON-DCM Trial	Hare JM *et al*. [65]	2017	Left ventricular function improved and scar size reduced in patients with dilated cardiomyopathy.
TAC-HFT Trial	Heldman AW *et al*. [66]	2014	Improvement in left ventricular ejection fraction (LVEF) was not significant.
MSC-HF Trial	Mathiasen AB *et al*. [67]	2015	Increased LVEF and left ventricular end-systolic volume (LVESV) were reduced in heart failure patients.
Heart Study	Yau TM *et al*. [68]	2018	Improvement in exercise capacity and cardiac function in heart failure patients with reduced ejection fraction.
REGENERATE-AMI Clinical Trial	Choudry F *et al*. [69]	2016	There is no significant improvement in primary endpoints for acute myocardial infarction.

## LIST OF ABBREVIATIONS

MSC: Mesenchymal Stem Cell
IHD: Ischemic Heart Disease
MI: Myocardial Infarction
VEGF: Vascular Endothelial Growth Factor
BMP-2: Bone Morphogenetic Protein-2
FGF-4: Fibroblast Growth Factor-4
HGF: Hepatocyte Growth Factor
cTnI: Cardiac Troponin I
MMP: Matrix Metalloproteases
BM-MSCs: Bone Marrow Mesenchymal Stem Cells
TNF-α: Tumor Necrosis Factor-α
IL: Interleukin
TGF-β: Transforming Growth Factor-β
Th1/Th2: T-Helper 1 / T-Helper 2
CVD: Cardiovascular Diseases
ECM: Extracellular Matrix
EV: Extracellular Vesicle
GMP: Good Manufacturing Practice
IFN-γ: Interferon-gamma
PGE2: Prostaglandin E2

## References

[1] Zhao Y., Xiong W., Li C. (2023). Hypoxia-induced signaling in the cardiovascular system: Pathogenesis and therapeutic targets.. Signal Transduct. Target. Ther..

[2] Paulino E.T. (2024). Development of the cardioprotective drugs class based on pathophysiology of myocardial infarction: A comprehensive review.. Curr. Probl. Cardiol..

[3] Mansouri F., Seyed Mohammadzad M.H. (2020). Molecular miR-19a in acute myocardial infarction: Novel potential indicators of prognosis and early diagnosis.. Asian Pac. J. Cancer Prev..

[4] Mansouri F., Seyed Mohammadzad M. (2020). Up-regulation of cell-free microRNA-1 and microRNA-221-3p levels in patients with myocardial infarction undergoing coronary angiography.. Adv. Pharm. Bull..

[5] Sadek H., Olson E.N. (2022). Toward the goal of human heart regeneration.. Dev. Cell.

[6] Deshmukh V., Wang J., Martin J.F. (2019). Leading progress in heart regeneration and repair.. Curr. Opin. Cell Biol..

[7] Jiang H., Fang T., Cheng Z. (2023). Mechanism of heart failure after myocardial infarction.. J. Int. Med. Res..

[8] Hussain M.S., Tyagi S., Khatri H., Singh S. (2022). Stem cell therapy for myocardial infarction: A mini-review.. Asian J Pharma Res Develop.

[9] Markov A., Thangavelu L., Aravindhan S. (2021). Retracted article: Mesenchymal stem/stromal cells as a valuable source for the treatment of immune-mediated disorders.. Stem Cell Res. Ther..

[10] Botleroo R.A., Bhandari R., Ahmed R. (2021). Stem cell therapy for the treatment of myocardial infarction: How far are we now?. Cureus.

[11] Renesme L., Pierro M., Cobey K.D. (2022). Definition and characteristics of mesenchymal stromal cells in preclinical and clinical studies: A scoping review.. Stem Cells Transl. Med..

[12] Fonseca L.N., Bolívar-Moná S., Agudelo T. (2023). Cell surface markers for mesenchymal stem cells related to the skeletal system: A scoping review.. Heliyon.

[13] Harrell C.R., Djonov V., Volarevic V. (2021). The cross-talk between mesenchymal stem cells and immune cells in tissue repair and regeneration.. Int. J. Mol. Sci..

[14] A-Elgadir TME, Shati AA, Alqahtani SA (2024). Mesenchymal stem cells improve cardiac function in diabetic rats by reducing cardiac injury biomarkers and downregulating JAK/STAT/iNOS and iNOS/Apoptosis signaling pathways.. Mol. Cell. Endocrinol..

[15] Poomani M.S., Mariappan I., Perumal R., Regurajan R., Muthan K., Subramanian V. (2022). Mesenchymal stem cell (MSCs) therapy for ischemic heart disease: A promising frontier.. Glob. Heart.

[16] Song N., Scholtemeijer M., Shah K. (2020). Mesenchymal stem cell immunomodulation: Mechanisms and therapeutic potential.. Trends Pharmacol. Sci..

[17] Yang G., Fan X., Liu Y. (2023). Immunomodulatory mechanisms and therapeutic potential of mesenchymal stem cells.. Stem Cell Rev. Rep..

[18] Hazrati A., Malekpour K., Khorramdelazad H., Rajaei S., Hashemi S.M. (2024). Therapeutic and immunomodulatory potentials of mesenchymal stromal/stem cells and immune checkpoints related molecules.. Biomark. Res..

[19] Huang Y., Wu Q., Tam P.K.H. (2022). Immunomodulatory mechanisms of mesenchymal stem cells and their potential clinical applications.. Int. J. Mol. Sci..

[20] Markmee R., Aungsuchawan S., Tancharoen W., Narakornsak S., Pothacharoen P. (2020). Differentiation of cardiomyocyte-like cells from human amniotic fluid mesenchymal stem cells by combined induction with human platelet lysate and 5-azacytidine.. Heliyon.

[21] Jiang H., Wang H., Liu T., Yang Z., Zhang R., Han H. (2018). Co-cultured the MSCs and cardiomyocytes can promote the growth of cardiomyocytes.. Cytotechnology.

[22] Song H., Li B., Guo R. (2022). Hypoxic preconditioned aged BMSCs accelerates MI injury repair by modulating inflammation, oxidative stress and apoptosis.. Biochem. Biophys. Res. Commun..

[23] Soltani L., Mahdavi A.H. (2022). Role of signaling pathways during cardiomyocyte differentiation of mesenchymal stem cells.. Cardiology.

[24] Farag A., Koung Ngeun S., Kaneda M., Aboubakr M., Tanaka R. (2024). Optimizing cardiomyocyte differentiation: Comparative analysis of bone marrow and adipose-derived mesenchymal stem cells in rats using 5-azacytidine and low-dose FGF and IGF treatment.. Biomedicines.

[25] Park B.W., Jung S.H., Das S. (2020). *In vivo* priming of human mesenchymal stem cells with hepatocyte growth factor–engineered mesenchymal stem cells promotes therapeutic potential for cardiac repair.. Sci. Adv..

[26] Guo Y., Yu Y., Hu S., Chen Y., Shen Z. (2020). The therapeutic potential of mesenchymal stem cells for cardiovascular diseases.. Cell Death Dis..

[27] Shafique S., Ali S.R., Rajput S.N., Salim A., Khan I. (2023). Cardiac transcription regulators differentiate human umbilical cord mesenchymal stem cells into cardiac cells.. Altern. Lab. Anim..

[28] Kumar R., Mishra N., Tran T., Kumar M., Vijayaraghavalu S., Gurusamy N. (2024). Emerging strategies in mesenchymal stem cell-based cardiovascular therapeutics.. Cells.

[29] Seow K.S., Ling A.P.K. (2024). Mesenchymal stem cells as future treatment for cardiovascular regeneration and its challenges.. Ann. Transl. Med..

[30] Chen Y., Shen H., Ding Y., Yu Y., Shao L., Shen Z. (2021). The application of umbilical cord‐derived MSCs in cardiovascular diseases.. J. Cell. Mol. Med..

[31] Raziyeva K., Smagulova A., Kim Y., Smagul S., Nurkesh A., Saparov A. (2020). Preconditioned and genetically modified stem cells for myocardial infarction treatment.. Int. J. Mol. Sci..

[32] Sid-Otmane C., Perrault L.P., Ly H.Q. (2020). Mesenchymal stem cell mediates cardiac repair through autocrine, paracrine and endocrine axes.. J. Transl. Med..

[33] Chen B., Chen X., Liu C., Li J., Liu F., Huang Y. (2018). Co-expression of Akt1 and Wnt11 promotes the proliferation and cardiac differentiation of mesenchymal stem cells and attenuates hypoxia/reoxygenation-induced cardiomyocyte apoptosis.. Biomed. Pharmacother..

[34] Borkowska P., Morys J., Zielinska A., Kowalski J. (2023). Effects of the Co-Overexpression of the *BCL* and *BDNF* Genes on the Gamma-Aminobutyric Acid-Ergic Differentiation of Wharton’s-Jelly-Derived Mesenchymal Stem Cells.. Biomedicines.

[35] Shen X., Pan B., Zhou H. (2017). Differentiation of mesenchymal stem cells into cardiomyocytes is regulated by miRNA-1-2 *via* WNT signaling pathway.. J. Biomed. Sci..

[36] Liu Z., Mikrani R., Zubair H.M. (2020). Systemic and local delivery of mesenchymal stem cells for heart renovation: Challenges and innovations.. Eur. J. Pharmacol..

[37] Ranganath S.H., Levy O., Inamdar M.S., Karp J.M. (2012). Harnessing the mesenchymal stem cell secretome for the treatment of cardiovascular disease.. Cell Stem Cell.

[38] Kastner N., Mester-Tonczar J., Winkler J. (2020). Comparative effect of MSC secretome to MSC co-culture on cardiomyocyte gene expression under hypoxic conditions *in vitro.*. Front. Bioeng. Biotechnol..

[39] Raman N., Imran S.A.M., Ahmad Amin Noordin K.B., Wan Kamarul Zaman W.S., Nordin F. (2022). Mechanotransduction of mesenchymal stem cells (MSCs) during cardiomyocytes differentiation.. Heliyon.

[40] Maacha S., Sidahmed H., Jacob S. (2020). Paracrine mechanisms of mesenchymal stromal cells in angiogenesis.. Stem Cells Int..

[41] Ahangar P., Mills S.J., Cowin A.J. (2020). Mesenchymal stem cell secretome as an emerging cell-free alternative for improving wound repair.. Int. J. Mol. Sci..

[42] Bhaskara M., Anjorin O., Wang M. (2023). Mesenchymal stem cell-derived exosomal micrornas in cardiac regeneration.. Cells.

[43] Song R., Dasgupta C., Mulder C., Zhang L. (2022). MicroRNA-210 controls mitochondrial metabolism and protects heart function in myocardial infarction.. Circulation.

[44] Schumacher D., Curaj A., Simsekyilmaz S., Schober A., Liehn E.A., Mause S.F. (2021). miR155 deficiency reduces myofibroblast density but fails to improve cardiac function after myocardial infarction in dyslipidemic mouse model.. Int. J. Mol. Sci..

[45] Khoei S.G., Dermani F.K., Malih S., Fayazi N., Sheykhhasan M. (2020). The use of mesenchymal stem cells and their derived extracellular vesicles in cardiovascular disease treatment.. Curr. Stem Cell Res. Ther..

[46] Li X., Guan Y., Li C., Zhang T. (2022). Immunomodulatory effects of mesenchymal stem cells in peripheral nerve injury..

[47] Stougiannou T.M., Christodoulou K.C., Dimarakis I., Mikroulis D., Karangelis D. (2024). To repair a broken heart: Stem cells in ischemic heart disease.. Curr. Issues Mol. Biol..

[48] Fu S.P., Wu X.C., Yang R.L. (2023). The role and mechanisms of mesenchymal stem cells regulating macrophage plasticity in spinal cord injury.. Biomed. Pharmacother..

[49] Weiss A.R.R., Dahlke M.H. (2019). Immunomodulation by mesenchymal stem cells (MSCs): Mechanisms of action of living, apoptotic, and dead msCs.. Front. Immunol..

[50] Wu X., Jiang J., Gu Z., Zhang J., Chen Y., Liu X. (2020). Mesenchymal stromal cell therapies: Immunomodulatory properties and clinical progress.. Stem Cell Res. Ther..

[51] Müller L., Tunger A., Wobus M. (2021). Immunomodulatory properties of mesenchymal stromal cells: An update.. Front. Cell Dev. Biol..

[52] Wang X., Tang Y., Liu Z. (2021). The application potential and advance of mesenchymal stem cell-derived exosomes in myocardial infarction.. Stem Cells Int..

[53] Sun X., Shan A., Wei Z., Xu B. (2018). Intravenous mesenchymal stem cell-derived exosomes ameliorate myocardial inflammation in the dilated cardiomyopathy.. Biochem. Biophys. Res. Commun..

[54] Wang X., Zhang H., Bai M. (2018). Exosomes serve as nanoparticles to deliver anti-miR-214 to reverse chemoresistance to cisplatin in gastric cancer.. Mol. Ther..

[55] Shao L., Zhang Y., Lan B. (2017). miRNA-sequence indicates that mesenchymal stem cells and exosomes have similar mechanism to enhance cardiac repair.. BioMed Res. Int..

[56] Zhao J., Li X., Hu J. (2019). Mesenchymal stromal cell-derived exosomes attenuate myocardial ischaemia-reperfusion injury through miR-182-regulated macrophage polarization.. Cardiovasc. Res..

[57] He J.G., Li H.R., Han J.X. (2018). GATA-4-expressing mouse bone marrow mesenchymal stem cells improve cardiac function after myocardial infarction *via* secreted exosomes.. Sci. Rep..

[58] Ma J., Zhao Y., Sun L. (2017). Exosomes derived from Akt-modified human umbilical cord mesenchymal stem cells improve cardiac regeneration and promote angiogenesis *via* activating platelet-derived growth factor D.. Stem Cells Transl. Med..

[59] Liu X., Li X., Zhu W. (2020). Exosomes from mesenchymal stem cells overexpressing MIF enhance myocardial repair.. J. Cell. Physiol..

[60] Feng Y., Huang W., Wani M., Yu X., Ashraf M. (2014). Ischemic preconditioning potentiates the protective effect of stem cells through secretion of exosomes by targeting Mecp2 *via* miR-22.. PLoS One.

[61] Makkar R.R., Price M.J., Lill M. (2005). Intramyocardial injection of allogenic bone marrow-derived mesenchymal stem cells without immunosuppression preserves cardiac function in a porcine model of myocardial infarction.. J. Cardiovasc. Pharmacol. Ther..

[62] Hare J.M., Traverse J.H., Henry T.D. (2009). A randomized, double-blind, placebo-controlled, dose-escalation study of intravenous adult human mesenchymal stem cells (prochymal) after acute myocardial infarction.. J. Am. Coll. Cardiol..

[63] Hatzistergos K.E., Quevedo H., Oskouei B.N. (2010). Bone marrow mesenchymal stem cells stimulate cardiac stem cell proliferation and differentiation.. Circ. Res..

[64] Hare J.M., DiFede D.L., Rieger A.C. (2017). Randomized comparison of allogeneic *versus* autologous mesenchymal stem cells for nonischemic dilated cardiomyopathy.. J. Am. Coll. Cardiol..

[65] Heldman A.W., DiFede D.L., Fishman J.E. (2014). Transendocardial mesenchymal stem cells and mononuclear bone marrow cells for ischemic cardiomyopathy: The TAC-HFT randomized trial.. JAMA.

[66] Mathiasen A.B., Qayyum A.A., Jørgensen E. (2015). Bone marrow-derived mesenchymal stromal cell treatment in patients with severe ischaemic heart failure: A randomized placebo-controlled trial (MSC-HF trial).. Eur. Heart J..

[67] Yau T.M., Pagani F.D., Mancini D.M. (2019). Intramyocardial injection of mesenchymal precursor cells and successful temporary weaning from left ventricular assist device support in patients with advanced heart failure.. JAMA.

[68] Choudry F., Hamshere S., Saunders N. (2016). A randomized double-blind control study of early intra-coronary autologous bone marrow cell infusion in acute myocardial infarction: The regenerate-AMI clinical trial.. Eur. Heart J..

[69] Sanz-Nogués C., O’Brien T. (2021). Current good manufacturing practice considerations for mesenchymal stromal cells as therapeutic agents.. Biomater Biosyst.

[70] Pakzad M., Hassani S.N., Abbasi F. (2022). A roadmap for the production of a gmp-compatible cell bank of allogeneic bone marrow-derived clonal mesenchymal stromal cells for cell therapy applications.. Stem Cell Rev. Rep..

[71] Ma C.Y., Zhai Y., Li C.T. (2024). Translating mesenchymal stem cell and their exosome research into GMP compliant advanced therapy products: Promises, problems and prospects.. Med. Res. Rev..

[72] Zhang R., Yu J., Mao Y., Zhang H. (2022). Efficacy and safety of mesenchymal stem cell therapy in patients with acute myocardial infarction: A systematic review and meta-analysis of randomized controlled trials.. Curr. Stem Cell Res. Ther..

[73] Shen T., Xia L., Dong W. (2021). A systematic review and meta-analysis: Safety and efficacy of mesenchymal stem cells therapy for heart failure.. Curr. Stem Cell Res. Ther..

[74] Giugni F.R., Giugni M.O.V., Pinesi H.T. (2024). Safety and efficacy of adipose-derived mesenchymal stem cell therapy for ischemic heart disease: A systematic review.. Arq. Bras. Cardiol..

[75] Matta A., Nader V., Lebrin M. (2022). Pre-conditioning methods and novel approaches with mesenchymal stem cells therapy in cardiovascular disease.. Cells.

[76] Ahmad B., Skorska A., Wolfien M. (2022). The effects of hypoxic preconditioned murine mesenchymal stem cells on post-infarct arrhythmias in the mouse model.. Int. J. Mol. Sci..

[77] Park E.H., Kim J.M., Seong E., Lee E., Chang K., Choi Y. (2023). Effects of mesenchymal stem cell injection into healed myocardial infarction scar border zone on the risk of ventricular tachycardia.. Biomedicines.

[78] Li M., Yamada S., Shi A. (2021). Brachyury engineers cardiac repair competent stem cells.. Stem Cells Transl. Med..

[79] Damasceno P.K.F., de Santana T.A., Santos G.C. (2020). Genetic engineering as a strategy to improve the therapeutic efficacy of mesenchymal stem/stromal cells in regenerative medicine.. Front. Cell Dev. Biol..

[80] Moeinabadi-Bidgoli K., Mazloomnejad R., Beheshti Maal A. (2023). Genetic modification and preconditioning strategies to enhance functionality of mesenchymal stromal cells: A clinical perspective.. Expert Opin. Biol. Ther..

[81] Zhang Y., Ravikumar M., Ling L., Nurcombe V., Cool S.M. (2021). Age-related changes in the inflammatory status of human mesenchymal stem cells: Implications for cell therapy.. Stem Cell Reports.

[82] Carp D.M., Liang Y. (2022). Universal or personalized mesenchymal stem cell therapies: Impact of age, sex, and biological source.. Cells.

